# Evaluation of the Potential Protective Effect of Ellagic Acid against Heavy Metal (Cadmium, Mercury, and Lead) Toxicity in SH-SY5Y Neuroblastoma Cells

**DOI:** 10.3390/foods13030419

**Published:** 2024-01-28

**Authors:** Rosanna Mallamaci, Alexia Barbarossa, Alessia Carocci, Daniela Meleleo

**Affiliations:** 1 Department of Biosciences, Biotechnologies and Environment, University of Bari “Aldo Moro”, 70125 Bari, Italy; rosanna.mallamaci@uniba.it; 2 Department of Pharmacy–Pharmaceutical Sciences, University of Bari “Aldo Moro”, 70125 Bari, Italy; alexia.barbarossa@uniba.it; 3 Department of Science of Agriculture, Food, Natural Resources and Engineering, University of Foggia, 71122 Foggia, Italy; daniela.meleleo@unifg.it

**Keywords:** antioxidants, heavy metals, neurotoxicity, SH-SY5Y, polyphenols

## Abstract

Ellagic acid (EA), a polyphenolic constituent of plant origin, has been thoroughly investigated for its hypothesised pharmacological properties among which antioxidant and neuroprotective activities are included. The present study was designed to explore whether EA could attenuate heavy metal (cadmium, mercury, and lead)-induced neurotoxicity in SH-SY5Y cells, which were utilized as a model system for brain cells. MTT and LDH assays were performed to examine the viability of the SH-SY5Y cells after exposure to Cd, Hg, and Pb (either individually or in combination with EA) as well as the effects of necrotic cell death, respectively. Furthermore, 2′,7′-dichlorodihydrofluorescein diacetate (DCFH-DA), a cell-based assay, was performed to determine whether EA could protect SH-SY5Y from heavy metal-induced oxidative stress. Results allowed us to assess the capability of EA to enhance the number of viable SH-SY5Y cells after exposure to heavy metal toxicity. Pre-treatment with EA showed a considerable, concentration-dependent, cytoprotective effect, particularly against Cd^2+^-induced toxicity. This effect was confirmed through the reduction of LDH release after the simultaneous cell treatment with Cd^2+^ and EA compared with Cd^2+^-treated cells. Furthermore, a significant, concentration-dependent decrease in reactive oxygen species (ROS) production, induced by H_2_O_2_ or heavy metals, was observed in the same model. Overall, the obtained results provide further insight into the protective role of EA against heavy metal-induced neurotoxicity and oxidative stress, thus indicating the potential beneficial effects of the consumption of EA-rich foods. However, to confirm its effects, well-designed human randomized controlled trials are needed to fill the existing gap between experimental and clinical research.

## 1. Introduction

Oxidative stress occurs when free radicals and other reactive species overwhelm the availability of antioxidants. Reactive oxygen species (ROS), reactive nitrogen species (RNS), and their counterpart antioxidant agents are essential for physiological signalling and host defence, as well as for the evolution and persistence of inflammation. However, when the intracellular sources of reactive species rise, alterations of several physiological processes occur. Among them, the increase in the blood–brain barrier’s permeability, tubulin alterations, and the perturbation in synaptic transmission are all included [1]. Oxidative stress has been linked to the aetiology of many diseases, including neurodegenerative diseases such as Alzheimer’s disease, amyotrophic lateral sclerosis, Friedreich’s ataxia, Huntington’s disease, multiple sclerosis, and Parkinson’s disease [2]. Among the substances that lead to ROS overproduction, a crucial role is played by heavy metals, which are also known to be associated with neurotoxicity and cognitive dysfunctions. These comprise cadmium (Cd), mercury (Hg), and lead (Pd) [3,4].

Cd, an environmental contaminant, is a toxic transition metal, which can induce several toxic effects depending on the concentration and the exposure time. The molecular mechanism for the toxic effects of Cd involves the alteration in thiol proteins [5], inhibition of energy metabolism [6], alteration in DNA structure [7], and an impact on some enzyme activities (succinate dehydrogenase, cyclic nucleotide phosphodiesterase, and antioxidant enzymes which include catalase, Mn-superoxide dismutase, Cu, and Zn-superoxide dismutase) [8,9,10]. Furthermore, increased levels of Cd are associated with very high toxicity of the kidneys, liver, lungs, testes, bones, blood system, and central nervous system [11]. Cd is reported to affect a number of cellular activities, such as cell proliferation, differentiation, and apoptosis, and is responsible for the development of several different tumour types [12,13]. Hg is a widespread environmental and industrial pollutant, which may cause severe tissue damage in different body districts [14]. Hg neurotoxic effects are well known [15]. Over the last decade, evidence has accumulated for the role of reactive oxygen metabolites as a mediator of tissue injury in several animal models [16]. The pro-oxidant properties of Hg have been documented over time [17]. Concerning the mechanism, research supports the view that Hg (II) ions both compromise the antioxidant potential of glutathione and promote the formation of reactive species through thiol complexation. Moreover, Hg induces lipid peroxidation [18]. Hg neurotoxicity seems to be linked to the induction of ROS and the depletion of antioxidant enzymes [19]. Pb is a well-known industrial and environmental toxin that can cause numerous acute and chronic toxicities. Indeed, its effects on the human body are devastating, and there is almost no function in the human body that is not affected by Pb toxicity. Particularly, long-term exposure in adults can result in anaemia along with an increase in blood pressure, severe damage to the brain and kidneys, miscarriage in pregnant women, and the reduction of male fertility [20]. Exposure to Pb appears to cause severe effects in cognitive function in both adults and children, among which intellectual and learning disabilities and behavioural disorders are included [21]. These deleterious effects are linked to several molecular and cellular mechanisms including alterations in DNA and chromosomal integrity [22]; Pb’s direct interaction with proteins, especially with those presenting metal-binding sites due to its high electronegativity; and the alteration to cellular redox status, considering that Pb can generate ROS simultaneously with the depletion of antioxidant systems due to its high affinity for thiol groups [23]. Indeed, Pb neurotoxicity is attributable to ROS generation and endogenous antioxidant reduction [24]. 

**Figure 1:**
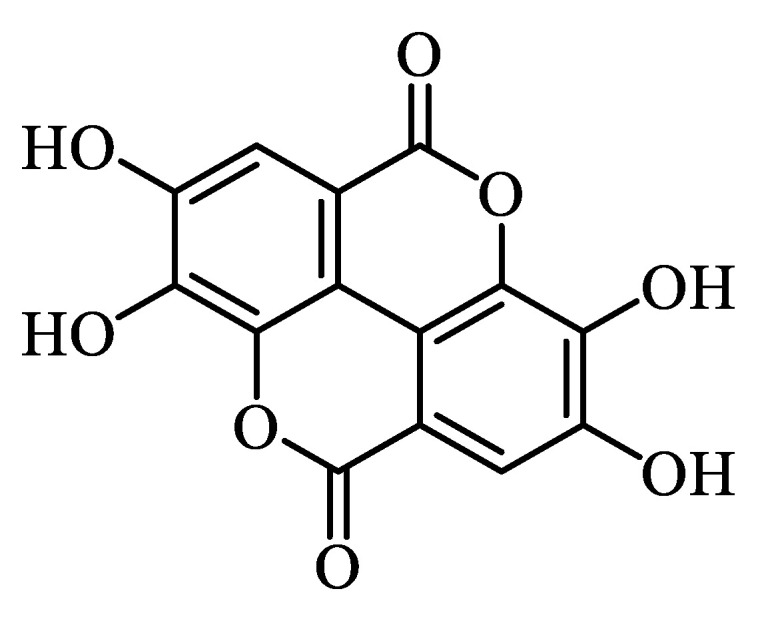
Ellagic acid.

Synthetic and natural antioxidants have been recognized in protecting against heavy metal-induced oxidative stress [25]. Among them, ellagic acid (EA), a polyphenolic compound (Figure 1), is a common metabolite found in several medicinal plants and vegetables including green tea, pomegranates, strawberries, blackberries, raspberries, nuts, grapes, and others [26]. It is present either in free-form or as part of more complex molecules (ellagitannins), the metabolism of which results in the release of EA as well as of several metabolites, including urolithins. EA has been reported to have anti-atherogenic, anti-inflammatory, and neuroprotective effects. Indeed, the health benefits attributed to EA-rich foods are thought to involve various protective mechanisms at the cellular level. Dietary EA is converted by the intestinal microbiota to urolithins, which are better absorbed than EA and may contribute significantly to the health effects attributed to EA-rich foods [27]. Among the mechanisms involved in EA pharmacological activities are its ability to reduce the lipidemic profile and lipid metabolism, alter pro-inflammatory mediators (tumour necrosis factor-α, interleukin-1β, interleukin-6), and decrease the activity of nuclear factor-κB while increasing nuclear factor erythroid 2-related factor 2 expression [28]. In addition, results from recent research have increased the interest in EA both as a potential protective agent of the liver [29] and skin [30] as well as as a potential anticancer agent due to the specific mechanisms affecting cell proliferation, apoptosis, DNA damage, and angiogenesis and its anti-inflammatory properties [31]. Several in vitro and in vivo studies have confirmed the protective role of EA in suppressing oxidative stress [32]. EA was found to scavenge ROS and RNS, such as hydroxyl radicals, perxoyl radicals, NO_2_ radicals, and peroxynitrite, with activities comparable to those of many well-known antioxidants such as vitamin E and vitamin C [33]. Indeed, the four hydroxyl and two lactone functional groups act, respectively, as hydrogen bond acceptors and donors, thus enabling EA to scavenge O_2_·^−^, HO·, H_2_O_2_, and ONOO· [34]. It has been reported that EA exerts antioxidant effects through Nrf2 and enhancing the expression of Heme oxygenase-1 and superoxide dismutase, as well as through the down-regulation of Keap1 in keratinocytes following UVA irradiation [35]. Numerous studies have reported the anti-oxidative benefits of foods enriched with EA, such as pomegranates, berries, and walnuts, on various metabolic diseases [36,37]. Moreover, it has been shown that EA increases the activity and expression levels of catalase, glutathione peroxidase, and glutathione-S-transferase [38]. Furthermore, some research has highlighted the neuroprotective potential of EA against the toxicity of certain agents, including rotenone and acrylamide, and the possible prevention of Alzheimer’s disease. [39,40,41]. Results of a recent study have shown that EA can alleviate the damaging effects of heavy metals, especially those of Pb, on the human body [42]. Oral EA treatment in rats has been shown to provide protection against Pb-induced nephrotoxicity by suppressing oxidative stress, inflammation, apoptosis, and autophagy [43]. EA reduces Pb residue levels in the kidneys, suggesting a chelating activity of this antioxidant against this metal. In addition, it has been demonstrated that the modulatory effect of EA against the metal-induced aggregation of α-Synuclein, which is an intrinsically disordered protein whose aggregation and deposition in Lewy bodies is involved in the progression of Parkinson’s disease and other related disorders [44]. Considering the high potential of EA in contributing to different aspects of health, in this study we aimed to investigate the potential neuroprotective effect of EA against heavy metals (Cd, Hg, and Pb) by counteracting both their cytotoxicity and the induction of oxidative stress in the SH-SY5Y cells used as a neuronal model. 

## 2. Results

### 2.1. Effect of Cd, Hg, Pb, and EA on Cell Viability

**Figure 2:**
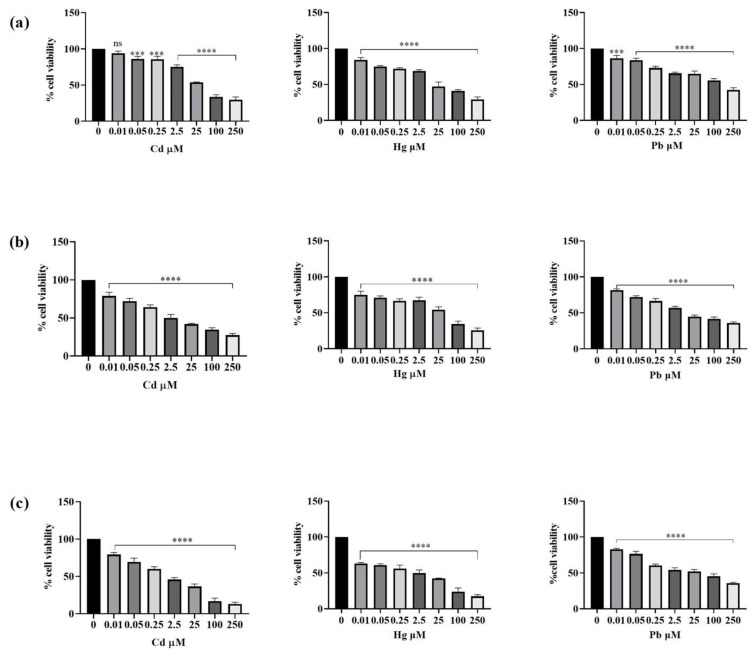
Effects of Cd^2+^, Hg^2+^, and Pb^2+^ (0.01–250 µM) on SH-SY5Y cell viability after 24 h (**a**), 48 h (**b**), and 72 h (**c**) of exposure. Results are shown as mean ± SEM *(n* = 3). Significant differences versus the control: non-significant differences (ns, *p* > 0.05), *** *p* < 0.001, **** *p* < 0.0001.

Cytotoxicity experiments were performed to determine the effect of Cd, Hg, and Pb on human SH-SY5Y neuronal cells. Results obtained are shown in Figure 2. The MTT findings demonstrated that metal concentration and time of exposure affect the cytotoxic effect on neuroblastoma cells. After 24 h of exposure, Cd^2+^ induced the most marked reduction in cell viability at the higher concentration used (250 µM) followed by Hg^2+^ and then Pb^2+^, which exhibited the lowest toxicity. In particular, the cytotoxicity of Cd^2+^ rose in a time-concentration manner. In fact, the cells’ survival rate dramatically decreased during 24 h at doses of 0.25, 2.5, 25, and 250 µM, decreasing from 86 to 75, 54, and 29%, respectively. Compared with Cd^2+^, Hg^2+^ was less toxic to SH-SY5Y cells. After 24 h, the survival rate dropped from 72% at 0.25 to 29% at 250 µM. Pb^2+^ cytotoxicity was the lowest among the three heavy metals, being quite consistent in the concentration range from 0.01 to 2.5 µM at all established times of treatment. Conversely, after 24 h, the survival rate decreased to 87% at 25 μM and 65% at 250 μM after Pb^2+^ exposure. Date obtained after 48 and 72 h of treatment confirmed what was observed in the 24 h experiment, i.e., Cd^2+^ was the metal most likely to strongly affect cell viability in a dose-dependent manner. The results obtained using the MTT assay were confirmed through observation of the cells under the inverted phase-contrast microscopy (see Supplementary Material, Figure S1). Indeed, cells in the control group, which were treated with the vehicle only, showed normal neuronal cell morphology after 24 h. On the contrary, cells exposed to Cd^2+^ at a concentration of 25 µM exhibited an irregular morphology, decreasing in number (about 50%) with respect to control cells. A similar cellular distribution and morphology were observed for cells treated with 25 µM Hg^2+^. Instead, after 24 h of treatment with Pb^2+^ cell morphology appeared more similar to that of the control cells with a slight decrease in the number of cells.

**Figure 3:**
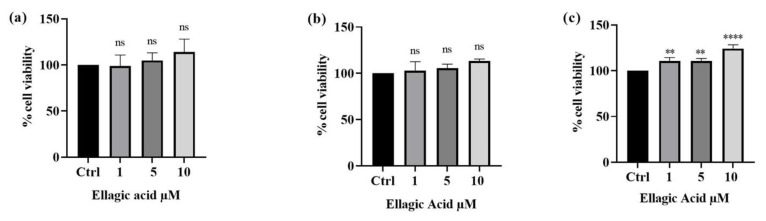
Effect of EA (1–10 µM) on SH-SY5Y cell viability after 24 h (**a**), 48 h (**b**), and 72 h (**c**) of exposure (MTT assay). Data are expressed as a percentage of vehicle-treated cells (control). Results are shown as mean ± SEM (n = 3). Significant differences versus the control: non-significant differences (ns, *p* > 0.05), ** *p* < 0.01, **** *p* < 0.0001.

The results from the first experimental set showed that a 24 h exposure time was sufficient to observe the harmful effects of the three metal ions, and that these effects dramatically increased with a longer exposure (72 h). These results supported the following toxicity scale for SH-SY5Y cells of Cd^2+^_,_ Hg^2+^, and then Pb^2+^. Additionally, the results obtained allowed us to choose the concentration of heavy metals which significantly decreased vitality for the evaluation of the potential protective action of the tested substance of EA. Therefore, a concentration of 25 µM was chosen for the successive experiments because, out of all the three metals, it was found to be the concentration capable of affecting cell viability without totally compromising the EA’s potential cytoprotective activity since it did not induce total cell death. Additionally, to assess the effect of EA on SH-SY5Y cells, the cellular line was exposed to 1, 5, and 10 µM concentrations of the compound for 24, 48, and 72 h. Cell viability was always higher at all concentrations with respect to the control. In particular, the results revealed a significant dose-dependent increase in cell viability compared with the control after 24, 48, and 72 h. In fact, after 24 h of exposure to EA, cell viability increased from 98.8% to 114.2%. Even more pronounced was the effect at the higher concentration (10 µM) which, at 72 h, produced an upturn in cell proliferation of 124.2% (Figure 3). Cell morphology results indicated a normal and uniform SH-SY5Y cell distribution along with an intact morphology after 24 h (see Supplementary Material, Figure S2). Cells exposed to different concentrations of EA (1–10 µM) showed a similar morphology at all the concentrations compared with the control cells (see Supplementary material, Figure S2, panel a). However, an increased number of cells has been observed, thus confirming the results obtained by means of the MTT assay.

### 2.2. Effect of EA on Cd-, Hg-, and Pb-Induced Cytotoxicity

**Figure 4:**
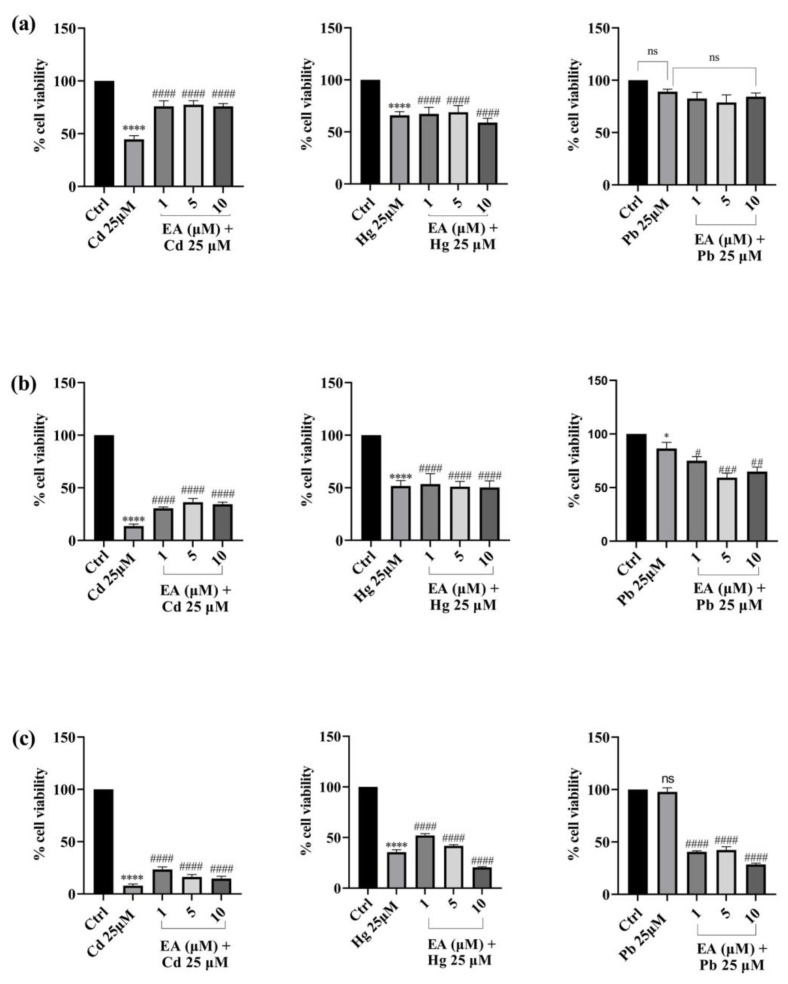
Effect of pre-treatment with Cd^2+^, Hg^2+^, and Pb^2+^ (25 µM) and EA (1–10 µM) on SH-SY5Y cell viability after 24 h (**a**), 48 h (**b**), and 72 h (**c**) of exposure (MTT assay). Data are expressed as mean ± SEM (n = 3). Significant differences versus the control: non-significant differences (ns, *p* > 0.05), * *p* < 0.05, **** *p* < 0.0001; # *p* < 0.05, ## *p* < 0.01, ### *p* < 0.001, #### *p* < 0.0001 as compared with Cd^2+^, Hg^2+^, or Pb^2+^ alone.

Once it had been established that the tested concentrations of EA were not toxic to the SH-SY5Y cell line, we proceeded to assess the possible cytoprotective effects against cell damage induced by exposure to Cd^2+^, Hg^2+^, and Pb^2+^ (25 μM) under various experimental conditions. Cells were first pre-treated with EA (1, 5, 10 µM) for a 12 h incubation period to test if it could protect cells from Cd^2+^, Hg^2+^, and Pb^2+^ toxicity (Figure 4). EA in pre-treatment exerted a marked, dose-dependent, cytoprotective effect against cells exposed to toxic concentrations of Cd^2+^. Indeed, the viability of cells pre-treated with various concentrations of EA was greater than the viability of cells treated with heavy metals alone. Particularly, cell viability values rise from a percentage of 46% (cells treated only with Cd^2+^) to 75.9% (cells pre-treated with 10 μM of EA) after 24 h of exposure. After 48 h of treatment, a significant increase was also noticed. In fact, the cell viability percentage improved from 13.7% (cells treated only with Cd^2+^) to 34.5% (cells pre-treated with 10 μM of EA). Regarding protection from Hg^2+^, slight improvements in cell viability compared with the controls were detected, at the EA concentrations of 1 and 5 μM, for all the incubation times. Conversely, no effect (after 24 h and 48 h of incubation) or even a slight reduction (after 72 h of incubation) of cell viability was observed at 10 μM. Unfortunately, pre-treatment with EA was not advantageous in Pb^2+^-treated cells at all EA concentrations after 24 and 48 h of exposure, while a decrease in viability was observed after 72 h of exposure. 

**Figure 5:**
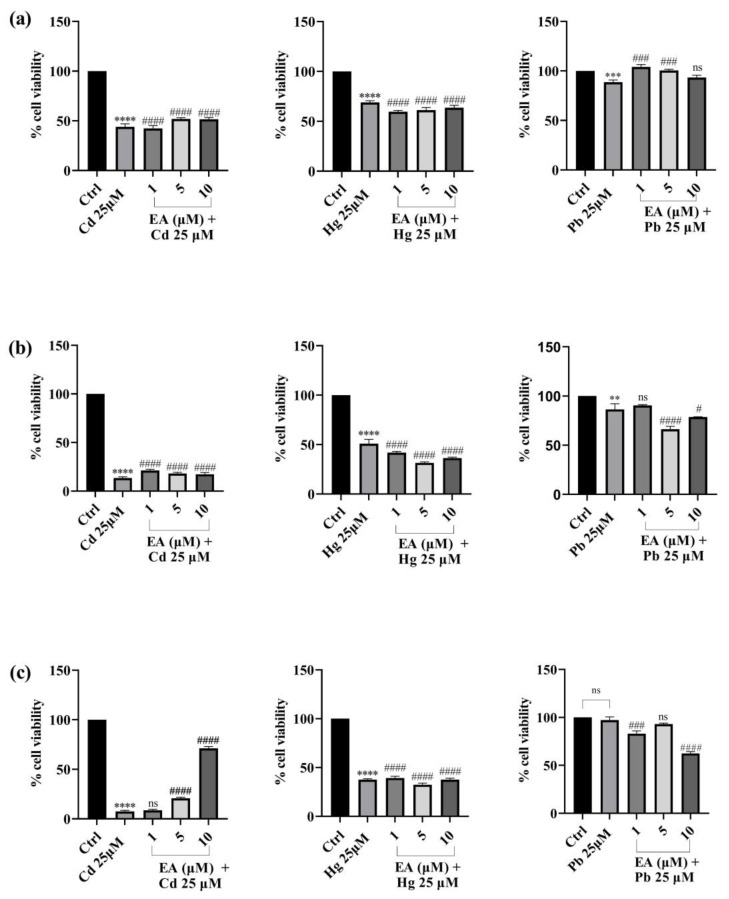
Effect of simultaneous treatment with Cd^2+^, Hg^2+^, and Pb^2+^ (25 µM) and EA (1–10 µM) on SH-SY5Y cell viability after 24 h (**a**), 48 h (**b**), and 72 h (**c**) of exposure (MTT assay). Data are expressed as mean ± SEM (n = 3). Significant differences versus the control: non-significant differences (ns, *p* > 0.05), ** *p* < 0.01, *** *p* < 0.001, **** *p* < 0.0001; # *p* < 0.05, ### *p* < 0.001, #### *p* < 0.0001 as compared with Cd^2+^, Hg^2+^, or Pb^2+^ alone.

Successively, the cytoprotective effect of the simultaneous treatment of EA with toxic concentrations of Cd^2+^ on cells was evaluated (Figure 5). In particular, the cell viability values increased from approximatively 46% (cells treated only with Cd^2+^) up to 53% (cells co-treated with 10 μM of EA) after 24 h of incubation. No significant cytoprotective effect was obtained for cells co-treated with EA and Hg^2+^. Encouraging results were instead obtained for cells treated simultaneously with EA and Pb^2+^. In fact, cell viability increased from 88.6% (Pb^2+^ only treated cells) to 104% in the case of co-treatment with EA 1 μM (24 h of exposure). A similar effect can be observed at higher concentrations of EA (5 or 10 μM) in the co-treatment. After 48 h of co-treatment, EA acid exhibited a pronounced cytoprotective effect against Cd^2+^-treated cells. Indeed, cell viability rose from 13% (cells treated only with Cd^2+^) to 20%. No cytoprotective effect was observed for the co-treatment between EA and Hg^2+^ or Pb^2+^. This trend of results was confirmed following 72 h of co-treatment.

### 2.3. Effect of EA on Cd^2+^-, Hg^2+^-, and Pb^2+^-Induced LDH Release

**Figure 6:**
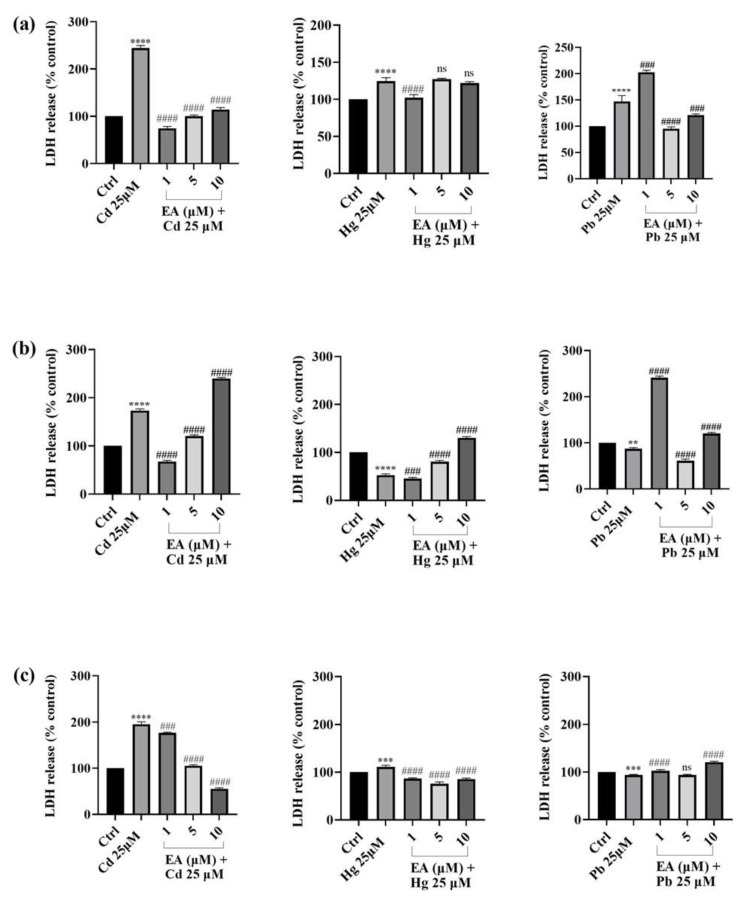
LDH activity of SH-SY5Y cell after of 24 h (**a**), 48 h (**b**), and 72 h (**c**) of exposure with different concentrations of EA (1–10 µM) and Cd^2+^, Hg^2+^, and Pb^2+^ (25 µM). Data are expressed as mean ± SEM (n = 3). Significant differences versus the control: non-significant differences (ns, *p* > 0.05), ** *p* < 0.01, *** *p* < 0.001, **** *p* < 0.0001; ### *p* < 0.001, #### *p* < 0.0001 as compared with Cd^2+^, Hg^2+^, or Pb^2+^ alone.

To explore the potential protective effect of EA against the oxidative stress-induced membrane damage trigged by heavy metals, a LDH assay was carried out on SH-SY5Y cells. This assay measures the activity of the stable enzyme LDH (lactate dehydrogetase) which is released into the medium because of apoptosis, necrosis, and other events of cell damage that cause membrane integrity decline [45] (Figure 6). Thus, the activity of LDH can be estimated and connected to the proportion of dead cells through cell lysis. Treatment with Cd^2+^, Hg^2+^, or Pb^2+^ alone significantly increased LDH release to 144%, 24%, and 47%, respectively, in SH-SY5Y after 24 h. When SH-SY5Y cells were exposed to mixtures of Cd^2+^/EA, LDH release significantly decreased after 24 h of treatment compared with Cd^2+^-treated cells at all tested EA concentrations. Regarding Hg^2+^/EA treatment, no significant reduction in LDH release was observed, while in the case of Pb^2+^, an appreciable decrease occurred during concomitant 5 and 10 μM of EA treatment after 24 h. Meanwhile, after 48 h of heavy metal treatment, an increase in LDH release was only observed for Cd^2+^, and the related decrease after Cd^2+^/EA co-treatment was obtained with 1 and 5 μM of EA. Interestingly, 72 h of Cd^2+^ treatment increased LDH release by 96%, while Cd^2+^/EA co-treatment significantly decreased LDH release in a clear-cut, dose-dependent manner up to 140% at 10 μM EA. 

### 2.4. ROS Scavenging Effects of EA against Cd^2+^-, Hg^2+^-, Pb^2+^-, and H_2_O_2_-Induced Oxidative Stress

**Figure 7:**
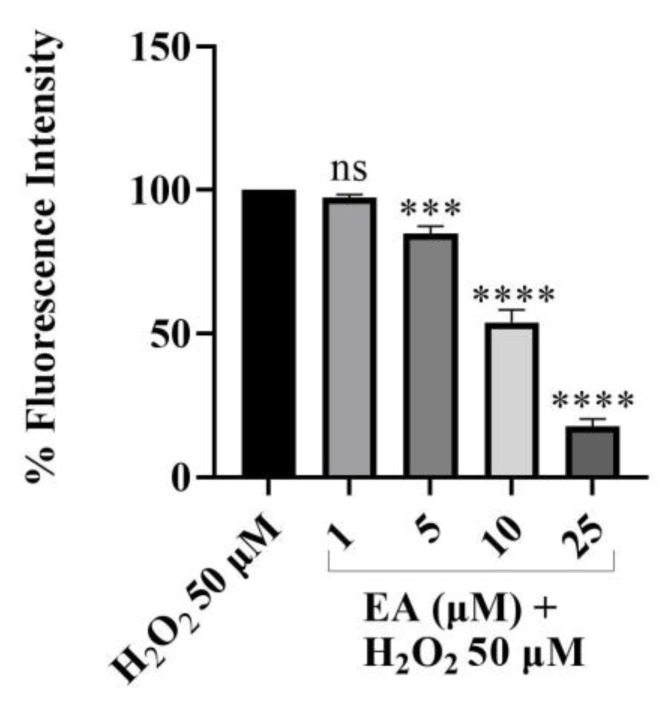
DCFH oxidation in SH-SY5Y cells after 24 h of incubation with increasing concentrations (1–25 µM) of EA in the presence of hydrogen peroxide (H_2_O_2_). Bars represent “medium ± standard deviation” values obtained from three individual experiments. Significant differences versus H_2_O_2_: non-significant differences (ns, *p* > 0.05), *** *p* < 0.001, **** *p* < 0.0001.

Since oxidative stress is one of the mechanisms of Cd^2+-^, Hg^2+^-, or Pb^2+^-induced neurodegeneration, we aimed at exploring the potential ability of EA to reduce oxidative stress triggered by heavy metals in SH-SY5Y cells. The potential antioxidant activity was evaluated in vitro using the 2′,7′-dichlorodihydrofluorescein diacetate (DCFH-DA) cell-based assay on SH-SY5Y cells. The test was based on measuring the reducing effect of the compound against oxidation of 2′,7′-dichlorodihydrofluorescein (DCFH) to the fluorescent probe of 2′,7′-dichlorofluorescein (DCF). At first, we determined the potential EA ability to protect our neuronal model from the ROS production induced by H_2_O_2_ (Figure 7), which was used at a concentration of 50 µM. The results demonstrated that EA reduced oxidative stress induced by H_2_O_2_ in a dose-dependent manner. In particular, at the higher concentration tested of 25 µM, pre-treatment with EA reduced the ROS production induced by H_2_O_2_ by 82.4%.

**Figure 8:**
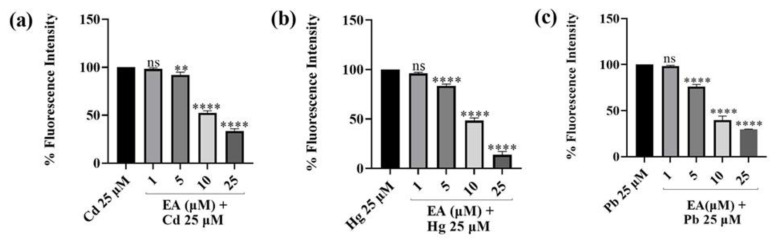
DCFH oxidation in SH-SY5Y cells after 24 h incubation with increasing concentrations (1–25 µM) of EA in the presence of Pb^2+^ (**a**), Cd^2+^ (**b**), or Hg^2+^ (**c**). Bars represent “medium ± standard deviation” values obtained from three individual experiments. Significant differences versus Cd^2+^, Hg^2+^, or Pb^2+^ alone: non-significant differences (ns, *p* > 0.05), ** *p* < 0.01, **** *p* < 0.0001.

Once we demonstrated the protective power of EA against H_2_O_2_-induced oxidation, we evaluated the same effect against Cd^2+^-, Hg^2+^-, and Pb^2+^-induced ROS production. Metals were used at a concentration of 25 µM (Figure 8). Moreover, in this case, the significant, concentration-dependent cytoprotective effect of EA could be observed against all the three metals. Although no remarkable antioxidant effect was detected at the lowest concentrations, pre-treatment with 10 and 25 µM of EA succeeded in decreasing Cd^2+^-induced oxidative stress by 47.6% and 66.4%, respectively. The most encouraging result was obtained against Hg^2+^-induced oxidation. Indeed, fluorescence intensity was reduced by 86.2% compared with Hg^2+^ treatment at the highest concentration. Regarding Pb^2+^, a slight protective effect has been detected at a concentration of 5 µM of EA, while EA conversely generated a marked decrease in oxidative stress at the highest concentration tested of 25 µM where the fluorescence intensity decreased by 70.4% with respect to cells treated only with Pb^2+^. 

## 3. Discussion

Environmental exposure to toxic heavy metals represents a widespread problem and poses a major risk to human health due to their long-lasting effects on the brain [46]. Evidence in support of the neurotoxic effects of Cd, Hg, and Pb exposure are increasing. Depending on the duration of the exposure, they may cause problems associated with neurodevelopment, neurobehaviour, and cognitive function, or they may contribute to the aetiology of age-related and neurodegenerative disorders such as Alzheimer’s (AD) and Parkinson’s (PD) diseases. Recently, it has been reported that Cd and Hg could affect the Aβ42 peptide, increasing its cytotoxicity, thus strengthening the hypothesis that they may be cofactors of the risk for AD [47]. Mitochondrial dysfunction, redox system imbalance, free radical-mediated generation of oxidative stress, decreasing antioxidative indices, interactions with protein sulfhydryl groups (-SH), and competition for essential metal binding sites (such as Fe, Cu, and Zn) are among the neurotoxic mechanisms of these heavy metals [48]. Additionally, cellular apoptosis, necrosis, and other cellular damage events can occur in response to Cd, Hg, and Pb toxicity [7,15,23]. Recently, the use of antioxidants to prevent or delay the adverse reactions produced by heavy metals has received great interest among researchers. Several reports indicate the possibility of using medicinal plant extracts comprising polyphenols, flavones, flavonoids, tannins, alkaloids, and terpenes, which are known antioxidants, as alternatives to neurotoxicity therapy [49]. EA is a polyphenolic compound that occurs naturally in numerous fruits, including strawberries, cranberries, pomegranates, grapes, and the seeds of raspberries. EA’s major therapeutic effects are linked to its anti-inflammatory, immunomodulatory, anti-diabetic, and anticancer properties [50]. Furthermore, EA has been reported to decrease oxidative stress-induced apoptosis by preventing caspase-3 activation through the mitochondrial apoptotic pathway in SH-SY5Y cells [51]. In addition, EA has been shown to have neuroprotective effects in Parkinson’s disease- and Alzheimer’s disease-affected experimental mice [52]. EA reduces lipid peroxidation, serves as a ROS scavenger, and positively affects mediators that are pro-oxidant and anti-inflammatory. Treatment with EA could inhibit Cd-induced oxidative injury in rat primary astrocytes [42]. EA dramatically boosted the activity of antioxidant enzymes, such as GPx, SOD, and CAT, in rats exposed to Hg. Additionally, in kidney tissue, EA increased the antioxidant system while simultaneously reducing the oxidant content [7]. In the present work, we attempt to demonstrate the cytoprotective ability of EA on SH-SY5Y neuroblastoma cells against heavy metal (Cd, Hg, and Pb) toxicity. 

### 3.1. Effect of Cd, Hg, Pb, and EA on Cell Viability

Cd^2+^, Hg^2+^, or Pb^2+^ were used to reproduce an in vitro model of heavy metal-induced toxicity in SH-SY5Y cells that could mimic an vivo heavy metal chronic exposure in the brain [53]. The MTT assay, a widely used test for cell viability determination, was performed on neuroblastoma cells in the presence of different concentrations of the single heavy metal. This way, we demonstrated that Cd^2+^, Hg^2+^, and Pb^2+^ significantly challenge cell viability in a dose- and time-dependent manner, with Cd being more toxic than Hg and Pb being the least lethal. The various processes used by SH-SH5Y cells to absorb heavy metals conceivably account for their different sensitivity to each heavy metal. Our findings confirmed what was previously reported. Indeed, Cd exposure has been shown to reduce cell viability in a dose-dependent manner and disrupts the cytoskeleton in cultured neuroblastoma cells, affecting signal transmission, cell function, and causing neurotoxicity [54]. Exposure to Pb significantly and concentration-dependently reduced the growth of SH-SY5Y cell line. The effects of Pb exposure were shown to induce changes to the Golgi apparatus, dysfunctional mitochondria, an increase in glial filaments in astrocytes, and oxidative stress. Pb further reduced glutathione levels by inactivating glutathione-S-transferase [55]. Extensive research showed the effect of methylmercury (MeHg) on SH-SY5Y cells, demonstrating that MeHg is able to damage SH-SY5Y cells in a dose- and time-dependent manner [56]. Our first experimental set allowed us to select the concentration of 25 μM, which significantly decreased cell vitality, to carry out subsequent experiments. Next, the investigation into the effect of EA on SH-SY5Y cells proved that EA did not negatively affect cell vitality and otherwise slightly improved their viability. Therefore, the concentrations of 1, 5, and 10 μM were selected to evaluate EA’s capability to counteract Cd^2+^-, Hg^2+0^-, or Pb^2+^-induced toxicity in SH-SY5Y. 

### 3.2. Effect of EA on Cd, Hg and Pb-Induced Cytotoxicity

Next, with the addition of EA to the cells treated with a toxic concentration of each heavy metal, the viability of the cells generally increased and a cytoprotective effect was seen. Pre-treatment with EA proved to be useful in protecting cells from Cd^2+^-induced toxicity, with the effects being more evident after 24 h of treatment with respect to 48 or 72 h of exposure. Whereas slight improvement or no effect in cell viability were detected when Hg^2+^ or Pb^2+^ induced the toxicity, respectively, simultaneous treatment with EA was shown to have a strong protective dose-dependent impact against Cd^2+^, particularly after 72 of treatment, while having a protective effect against Pb^2+^ at 24 h and no effect against Hg^2+^. 

### 3.3. Effect of EA on Cd^2+^-, Hg^2+^-, and Pb^2+^-Induced LDH Release

One of the most common types of physiologically recognized cell death is necrosis. To investigate the impact of neurotoxic substances on cell death, LDH leakage, a general marker of cell membrane damage and necrotic cell death, can be measured [57]. Indeed, cells treatment with Cd^2+^, Hg^2+^, or Pb^2+^ at concentrations that resulted in a considerable decline in cell viability induced significant LDH leakage, thus showing that the induction of necrosis was one of the main drivers of the reduction in cell viability. Therefore, in our study, the LDH assay, which was performed on SH-SY5Y exposed to the heavy metals and in the presence of EA, confirmed the same trend of results obtained using the MTT assay. Indeed, when SH-SY5Y cells were exposed to mixtures of Cd^2+^/EA, a significant decrease in enzyme release was observed after 24 h of treatment compared with Cd^2+^-treated cells, thus demonstrating that EA significantly reduced the negative effects caused by 25 µM the heavy metal. The most interesting results was observed after 72 h of treatment when a stronger dose-dependent reduction in the enzyme release was observed. Conversely, no noteworthy effect of the simultaneous treatment with EA and Hg^2+^ was possible to observe for LDH release because of the slight rise in LDH release that was induced by Hg^2+^ only at 24 h. Finally, with regard to Pb^2+^, a good EA protective effect was detected after 24 h of treatment. Our findings allow us to assess that Cd^2+^-, Hg^2+^-, and Pb^2+^-induced toxicity causes cell death and, almost as observed with regard to Cd^2+^, this occurs most likely through necrosis as suggested by the results of the LDH assay. However, the treatment of cells with EA inhibits LDH release and particularly shields SH-SY5Y cells from Cd^2+^ neurotoxicity. 

### 3.4. ROS Scavenging Effects of EA against Cd^2+^-, Hg^2+^-, Pb^2+^-, and H_2_O_2_-Induced Oxidative Stress

Furthermore, we aimed at exploring the neuroprotective effects of EA demonstrating its ability to reduce oxidative stress induced by heavy metals in SH-SY5Y cells, thus confirming that EA may act as a ROS scavenger and as a regulator of antioxidant systems. We firstly assessed that EA was able to protect our neuronal model from ROS production induced by stimulus with H_2_O_2_ in a dose-dependent manner. The same effect was demonstrated against Cd^2+^-, Hg^2+^-, and Pb^2+^-induced ROS production. Indeed, EA was shown to be effective in counteracting ROS production induced by these metals, since a significant and concentration-dependent cytoprotective effect could be observed, the most encouraging result being obtained for Hg^2+^-induced oxidative stress protection. The obtained outcomes conceivably could be due to the already proven capabilities of EA to chelate metals, to act as radical scavenger and to promote cellular antioxidant enzymes activity [58].

## 4. Materials and Methods

### 4.1. Chemicals and Reagents

EA powder was purchased from Farmalabor srl (Canosa di Puglia, Italy). CdCl_2_, HgCl_2,_ or Pb(NO_3_)_2_, high glucose (4.5 gL^−1^) Dulbecco’s modified Eagle medium (DMEM), fetal bovine serum (FBS, PAN Biotech, Aidenbach, Germany), L-glutamine, trypsin, MTT, LDH, and 2′,7′-dichlorofluorescein diacetate were purchased from Sigma-Aldrich S.p.a. (Milan, Italy).

### 4.2. Culture Cells

The human SH-SY5Y neuroblastoma cells used in this study were purchased from the American Type Culture Collection (ATCC, Manassas, VA, USA). The cells were normally cultured in 25 cm^2^ flasks (Corning Inc., New York, NY, USA) and maintained in high glucose (4.5 gL^−1^) Dulbecco’s modified Eagle medium (DMEM), supplemented with 10% (*v*/*v*) fetal bovine serum (FBS, PAN Biotech), 4 mM of L-glutamine (Sigma-Aldrich S.p.a. Milan, Italy)), and 1% (*v*/*v*) antibiotic solution penicillin-streptomycin (LONZA Bioscience, Walkersville, MD, USA). The cells were maintained at 37 °C in an incubator (Thermo Scientific Hera Cell 240i, Waltham, MA, USA) with 5% CO_2_ in the air atmosphere and 95% relative humidity. Complete growth medium (DMEM) was changed every two days. After being 80% confluent, the cells were washed with phosphate-buffered saline solution (PBS) to remove any unattached cells. The attached cells were harvested using a 1 mL of 0.25% trypsin and 0.53 mM of EDTA solution (SIGMA) and then seeded at a density of 5 × 10^3^ cells/well in a 96-well plate and incubated for 24 h to allow for attachment.

### 4.3. Determination of Cell Viability

The MTT (3-(4,5-dimethylthiazol-2-yl)-2,5- diphenyltetrazolium bromide) technique, which relies on the ability of mitochondrial oxidoreductases to convert soluble MTT into insoluble formazan in live cells, was used to determine the vitality of the cells. The amount of formazan generated corresponds to the number of live cells [59]. In brief, SH-SY5Y cells were seeded at the density of 5 × 10^4^ in a 96-well plate (6 wells/concentration group plus 1 control group). To monitor the toxicity of Cd^2+^, Hg^2+^, or Pb^2+^ on SH-SY5Y neuroblastoma cells, we prepared the following experimental sets: in the first experimental set, cells were exposed to increasing concentrations of three heavy metals Cd^2+^, Hg^2+^, or Pb^2+^ (0.01. 0.05, 0.25, 2.5, 25, and 250 μM) in 6 wells per concentration group, and the cells were incubated for 24, 48, and 72 h. In the second set of experiments, cells were pre- or co-treated with increasing concentrations of EA (1, 5, and 10 μM) and incubated for 24, 48, and 72 h. Control groups consisted of SH-SY5Y cells, which were processed in the same manner and incubated simultaneously as the treated groups. Subsequently, cells were incubated with 20 μL of MTT stock solution (5 mg/mL in PBS 1X) in 180 μL of medium in the dark. After an additional three hours at 37 °C, the medium was taken out, 150 μL of DMSO was added to dissolve the formazan crystals, and the mixture was kept warm for five minutes while being stirred. The absorbance was recorded at 540 nm with a multilabel microplate reader Victor 3 (PerkinElmer, Waltham, MA, USA). All MTT assays were performed in triplicate. Cell viability is expressed as a percentage of the control group (% control) calculated from the equation: *% control = Absorbance treatment/Absorbance control × 100%.* Data are the mean percentages of viable cells vs. the respective controls. A DMSO medium was used for control cells.

### 4.4. Lactate Dehydrogenase (LDH) Release

Lactate dehydrogenase (LDH) leakage into the culture medium was used to measure cytotoxicity. To get a cell-free supernatant, media were sucked and centrifuged for 5 min. at 3000 rpm after being exposed to the Cd^2+^, Hg^2+^, or Pb^2+^ and EA. The supernatant was used for the assay on LDH activity. Briefly, the reaction was initiated by mixing 0.1 mL of cell free medium with 48 mM of potassium phosphate buffer (pH 7.5) containing 0.18 mM of NADH and 0.6 mM of sodium pyruvate in a final volume of 3 mL. A microplate spectrophotometer system (Bio-Rad-680, Bio-Rad, Redmond, WA, USA) was used to measure the change in absorbance at 440 nm. The formula for calculating cell LDH release (% control) is as follows: % control = (U LDH/mg cell protein) treatment/(U LDH/mg cell protein) control 100% [60,61]. A DMSO medium was used for control cells.

### 4.5. Measurement of Intracellular ROS Production

According to a slightly modified procedure already reported in the literature, the generation of ROS was determined using an oxidation-sensitive fluorescent probe, 2′,7′- dichlorodihydrofluorescein diacetate [62]. Briefly, the SHSY-5Y cells were seeded into a 96-black well plate for 24 h. After pretreatment with the indicated concentrations of EA (0, 1, 5, 10, and 25 μM) for 24 h, cells were treated with Cd^2+^, Pb^2+^, or Hg^2+^ (25 μM) for 6 h or H_2_O_2_ (50 µM) for 30 min. Then, they were tested using a fluorescent probe (DCFH-DA) and added in the dark and incubated at 37° for 30 min. The formation of fluorescent dichlorofluorescein (DCF) due to oxidation of DCFH in the presence of ROS was read at 530 nm using a microplate reader Tecan Infinite M1000 Pro (Tecan, Cernusco S.N., Italy) and a DMSO medium was used for control cells.

### 4.6. Statistical Analysis 

All the results are expressed as mean ± standard deviation (SD). The reported values are the results of at least three experiments. All assays were performed in triplicate to ensure reproducibility. Data were analysed for statistical significance (*p* < 0.0001) using one-way ANOVA followed by Dunnett’s test performed through GraphPad Prism 9.0. A *p* value ≤ 0.05 was considered significant.

## 5. Conclusions

The findings of this work demonstrate that exposure to heavy metals such as Cd, Hg, and Pb damages SH-SY5Y cells causing a concentration- and time-dependent reduction i their viability, with cells death conceivably occurring through necrosis. Despite acting in varying concentrations, EA showed to have high protective activity against SH-SY5Y cells exposed to Cd, Hg, or Pb toxicity, counteracting the cytotoxic effects of these metals in the proposed cellular model. The toxicity that was identified through the MTT assay and the measurement of LDH release was reversed through treatment with EA, which offers further evidence that EA can have a positive impact on neuronal cells protection. This cytoprotective activity was particularly evident against the highly toxic heavy metal Cd, whose neurotoxicity has been widely documented over the years. Additionally, our study showed how effective EA is at reducing oxidative stress provoked through stimulus with both H_2_O_2_ and heavy metals, with this effect being more pronounced against Hg-induced oxidative stress. Although further investigation will be needed to clarify the precise mechanism behind these effects of EA, these results could contribute to drawing attention on EA as a promising candidate to be employed as a natural food resource for neuroprotection.

## Data Availability

Data is contained within the article and supplementary material.

## Supplementary Materials

The following supporting information can be downloaded at: https://www.mdpi.com/article/10.3390/foods13030419/s1, Figure S1: Inverted phase-contrast images of SH-SY5Y cells (20× magnification) treated with only-vehicle, control cells (a), Cd^2+^ (b), Hg^2+^ (c), and Pb^2+^ (d) at a concentration of 25 µM; Figure S2: Inverted phase-contrast images of SH-SY5Y cells (20× magnification) treated with only-vehicle, control cells (a), EA 1 µM (b), EA 5 µM (c), and EA 10 µM (d)
