# Supplementary material for: Evaluation of the Potential Protective Effect of Ellagic Acid against Heavy Metal (Cadmium, Mercury, and Lead) Toxicity in SH-SY5Y Neuroblastoma Cells
Source: Foods. 2024 Jan 28;13(3):419. doi: 10.3390/foods13030419 (PMC10855963; doi:10.3390/foods13030419)
Supplement: Supplementary file 1 [file foods-13-00419-s001.zip › foods-2813333-supplementary.pdf]

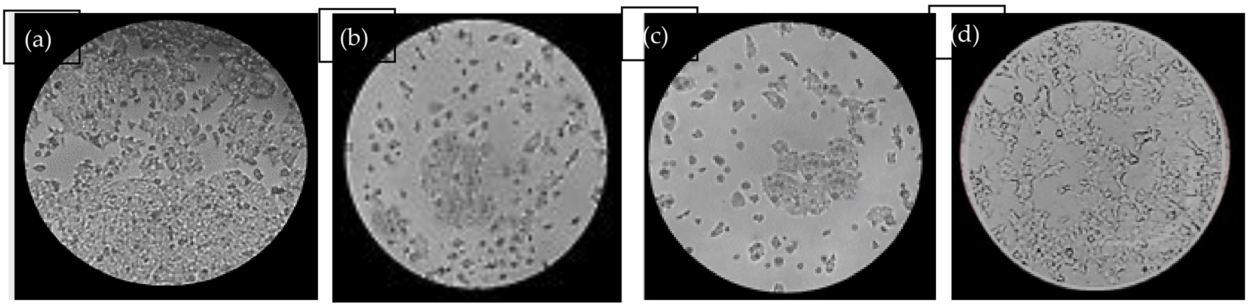

Figure S1. Inverted phase-contrast images of SH-SY5Y cells (20 x magnification) treated with only-vehicle, control cells (a), Cd^{2+} (b), Hg^{2+} (c), and Pb^{2+} (d) at a concentration of 25 μM.

The results obtained with the MTT assay were confirmed by the observation of the cells under the inverted phase-contrast microscopy (Figure 1). Indeed, cells in the control group, treated with the vehicle only, showed normal neuronal cells morphology after 24h. On the contrary, cells exposed to Cd^{2+} at a concentration of 25 μM exhibited an irregular morphology, decreasing in number (about 50%) with respect to control cells. A similar cellular distribution and morphology was observed for cells treated with Hg^{2+} 25 μM. Instead, after 24h of treatment with Pb^{2+} cells morphology appeared more similar to that of control cells with a slight decrease in cells number.

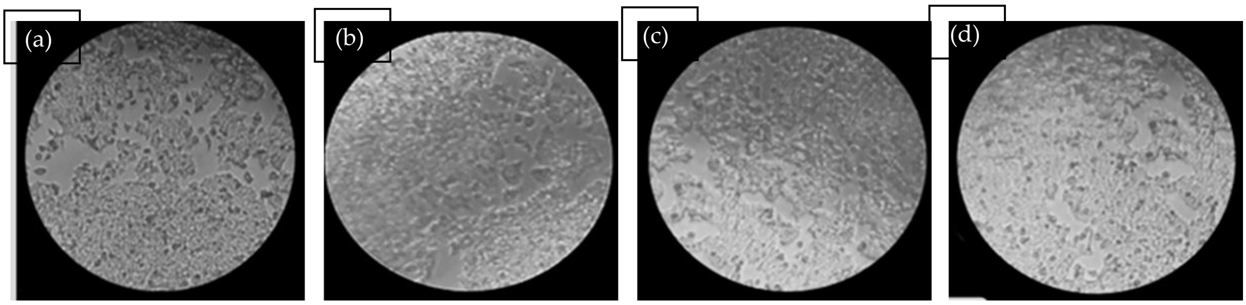

Figure S2. Inverted phase-contrast images of SH-SY5Y cells (20 x magnification) treated with only-vehicle, control cells (a), EA 1 μM (b), EA 5 μM (c), and EA 10 μM (d).

Cell morphology results indicated a normal and uniform SH-SY5Y cells distribution along with an intact morphology after 24h. Cells exposed to different concentrations of EA (1–10 μM) showed a similar morphology at all the concentrations compared to control cells (a). However, an increased cells number has been observed thus confirming the results obtained by means of the MTT assay.
